# CAFuncAPA: a knowledgebase for systematic functional annotations of APA events in human cancers

**DOI:** 10.1093/narcan/zcad004

**Published:** 2023-01-23

**Authors:** Kexin Huang, Sijia Wu, Xiaotong Yang, Tiangang Wang, Xi Liu, Xiaobo Zhou, Liyu Huang

**Affiliations:** School of Life Science and Technology, Xidian University, Xi’an, Shaanxi, P.R. China; West China Biomedical Big Data Centre, West China Hospital, Sichuan University, Chengdu, Sichuan 610041, P.R. China; School of Life Science and Technology, Xidian University, Xi’an, Shaanxi, P.R. China; School of Life Science and Technology, Xidian University, Xi’an, Shaanxi, P.R. China; School of Life Science and Technology, Xidian University, Xi’an, Shaanxi, P.R. China; School of Life Science and Technology, Xidian University, Xi’an, Shaanxi, P.R. China; Center for Computational Systems Medicine, School of Biomedical Informatics, The University of Texas Health Science Center at Houston, Houston, TX 77030, USA; McGovern Medical School, The University of Texas Health Science Center at Houston, Houston, TX, USA; School of Life Science and Technology, Xidian University, Xi’an, Shaanxi, P.R. China

## Abstract

Alternative polyadenylation (APA) is a widespread posttranscriptional regulation process. APA generates diverse mRNA isoforms with different 3’ UTR lengths, affecting mRNA expression, miRNA binding regulation and alternative splicing events. Previous studies have demonstrated the important roles of APA in tumorigenesis and cancer progression through diverse aspects. Thus, a comprehensive functional landscape of diverse APA events would aid in a better understanding of the underlying mechanisms related to APA in human cancers. Here, we built CAFuncAPA (https://relab.xidian.edu.cn/CAFuncAPA/) to systematically annotate the functions of 15478 APA events in human pan-cancers. Specifically, we first identified APA events associated with cancer survival and tumor progression. We annotated the potential downstream effects of APA on genes/isoforms expression, regulation of miRNAs, RNA binding proteins (RBPs) and alternative splicing events. Moreover, we also identified up-regulators of APA events, including the effects of genetic variants on poly(A) sites and RBPs, as well as the effect of methylation phenotypes on APA events. These findings suggested that CAFuncAPA can be a helpful resource for a better understanding of APA regulators and potential functions in cancer biology.

## INTRODUCTION

Alternative polyadenylation (APA) is a widespread posttranscriptional regulation process that occurs on >70% of human genes (1). APA generates diverse transcript isoforms with different 3’ untranslated regions (3’UTRs) through cleavage or polyadenylation at multiple polyadenylation sites (PAS) (1). Previous studies have demonstrated the important roles of APA in mRNA metabolism, export, stability and translation efficiency (2). In addition, APA also showed potential effects in multiple biological processes, such as microRNA (miRNA) binding regulation, alternative splicing (AS), and RNA binding protein (RBP) regulation (3). In cancer studies, APA has been demonstrated to be involved in tumorigenesis and cancer progression (4). For example, proto-oncogenes prefer to use proximal poly(A) sites to generate shortened 3’UTRs, leading to miRNA dysregulation of these genes, such as insulin-like growth factor 2 mRNA-binding protein 1 (IGF2BP1). The shorter isoform of IGF2BP1 eliminates let-7 miRNA binding, thus resulting in a more profound oncogenic transformation in cancer cells (5). Thus, the development of a functional reference of APA events across cancers will provide knowledge of the potential mechanism in cancer and identify potential targets for cancer treatment strategies.

To our knowledge, there are two databases (TC3A and SNP2APA) providing APA annotations in pan-cancers (6,7). TC3A provides an APA usage landscape in multiple cancers, SNP2APA focus on identification of the single-nucleotide polymorphism (SNPs) that potentially affect APA events in cancer using quantitative trait locus (QTL) analysis. However, due to the complex and multiple regulation function of APA, our knowledge of APA modulation in biological processes, such as miRNA and RBP regulation in cancer remains incomplete. Here, we built CAFuncAPA, a knowledgebase of a systematic functional annotation of APA events in human cancer. Our annotation including APA up-regulator analysis, such as the effect of the SNPs located in PAS and RBPs on APA events, and the effect of methylation phenotypes on APA events. To better understand the potential effects of the tumor-associated APA events, the down-stream effects included the analyses of APA effects on genes/isoforms expression, miRNA binding regulation, RBP regulation and alternative splicing regulation. These annotations will be helpful to understand the APA modulation in multiple biological processes. Supplementary Table S1 shows the comparison between CAFuncAPA and previous APA databases. CAFuncAPA is the first and unique database that holistically annotates APA functions in human cancer from diverse aspects.

In the present study, we first collected 15478 APA events across 32 cancer types from the TC3A database. Based on these data, we first identified the APA events related to cancer survival and tumor progression. To better understand the potential mechanisms of these tumor-associated APA events, we performed APA downstream effect and APA up-regulators analyses. The down-stream effects included the analyses of APA effects on genes/isoforms expression, miRNA binding regulation, RBP regulation and alternative splicing regulation. The APA up-regulators analyses included the effect of the genetic variants on PAS and RBPs, and the effect of methylation phenotypes on APA events. We also identified gene–drug interactions for APA genes. We hope that CAFuncAPA will be helpful for a better understanding of APA regulation on oncogenes in cancer biology.

## MATERIALS AND METHODS

### Data download and collection

To explore the functional mechanism of APA events, we first downloaded APA event information (percentage of distal poly(A) site usage index, PDUI) of 32 cancer types from download section of TC3A website (http://tc3a.org) (6). We also downloaded gene expression profiles, SNP genotype, methylation phenotype, and clinical data (patient survival and pathological stage information) from TCGA using ‘TCGAbiolink’ package (v3.16) in R (8). The isoform expression profiles for each gene were download from TCGA by using firebrowse (http://firebrowse.org/). miRNAs and target genes were obtained from TargetScan Human 8.0 website (https://www.targetscan.org/vert_80/) (9). RBP and targeted mRNA pairs were downloaded from starBase v2.0 website (http://starbase.sysu.edu.cn) (10). Splicing factors (SFs) and alternative splicing event information of 32 cancer types were collected from collected from SpliceAid-F database (http://srv00.recas.ba.infn.it/SpliceAidF/) and previous pan-cancer studies (11–16). SNPs and their location information were downloaded from dbSNP (version: Build 151) (https://www.ncbi.nlm.nih.gov/projects/SNP/snp_summary.cgi) (17). The data source, download tools were summarized in the Supplementary Table S2.

### Identification of prognosis- and stage-associated APA events

The annotations of APA events started from the identification of prognosis- and stage-associated APA events in 32 cancer types. We divided the patients into two groups according to the median PDUI. Then, we performed both Kaplan–Meier (K–M) survival analysis and Cox survival analysis for each APA event gene using the ‘survival’ package in R (18). We also performed ANOVA analysis to compare APA events between different tumor pathology stages in each cancer type using ‘aov’ function in R. Only APA events with a significant association (*P* < 0.05 and Num > 30) with prognosis and tumor stages were retained in the database.

### Correlation analysis between APA events and gene/isoform expression

Previous studies have demonstrated that APA regulates gene expression through generating various isoforms with different 3’UTR lengths (19). Here, we first identified APA events that potentially occur on gene isoforms. Then, to identify APA regulation of gene expression and isoform expression, we performed Pearson correlation analysis between tumor-associated APA events and gene/isoform expression. Correlation analysis was performed using the ‘cor’ function in R. During the analysis, only APA events showing a significant correlation (*P* < 0.05 and Num > 30) with their mRNA or isoform expression were retained in the database. Moreover, to identify the prognostic and staging association of APA-regulated expression, we also performed survival analysis and ANOVA analysis between different stages using gene/isoform expression. The threshold was same as previous.

### Identification of APA-regulated miRNA escape

APA-medicated shortening of the 3’UTR may lead to escape of miRNA regulation through loss of miRNA binding sites (20). To better understanding the effects of APA on miRNA regulation, we identified miRNA binding sites that were located after APA sites using TargetScan. These miRNAs were identified as escaped miRNAs. Moreover, we also identified potential target genes that may be regulated by escaped miRNAs. Target gene list of escaped miRNAs was downloaded from TargetScan. Correlation analyses among PDUI, APA gene expression and miRNA target gene expression were performed using R. The significance threshold of correlation analysis was set as *P* < 0.05 and Num > 30. Moreover, we also performed QTL analysis between APA events and miRNA target gene expression using additive linear model ultra-fast eQTL analysis via large matrix operations (MatrixEQTL) in R. We first performed quality control for the expression data and the APA events data (PDUI). The expression data and the APA events data was excluded if: (i) the rate of NA values is >95% and (ii) the standard deviation between samples of a given gene is <0.05. The significance was set as $P < 1 \times {{\mathrm{e}}}^{ - 5}$ for QTL analysis. Then we selected miRNA target genes from significant APA-gene-expression pairs. The age and sex of patients were set as covariates. To obtain more reliable results, we only left the overlap results of two methods in the database. Details of the methods were summarized in the Supplementary Table S3.

### Identification of the effect of APA events in RBP genes on their targets

APA events in RBP genes may lead to altered expression of RBP targets (4). Here, we obtained RBPs and their targets from starBase, and performed interaction analysis between APA events in RBP genes and RBP targets (10). Correlation analyses were performed between APA events and RBP gene expression, APA events and RBP target expression, and RBP gene expression and RBP target expression. The threshold of significant correlations was set as *P* < 0.05 and Num > 30. We also performed QTL analysis between APA events and RBP target genes using additive linear model to identify genes that potentially associated with APA events in RBP. The expression data and the APA events data was excluded if: (i) the rate of NA values is >95% and (ii) the standard deviation between samples of a given gene is <0.05. The significance was set as $P < 1 \times {{\mathrm{e}}}^{ - 5}$. Then we selected RBP target genes from significant APA-gene-expression pairs. The age and sex of patients were set as covariates. Only the overlap results of two methods will be left in the database. Details of the methods were summarized in the Supplementary Table S3.

### Identification of the effect of APA events in splicing factors on alternative splicing

A prior study has found that APA occurring SFs may affect AS (21). Thus, we performed interaction analysis between APA events in SFs and splicing events. Correlation analysis between APA events in SFs and splicing events on targets was performed. The interactions with significant correlations (*P* < 0.05 and Num > 30) were selected. Moreover, the QTL analysis was performed for APA events and alternative splicing events using additive linear model. The alternative splicing data and the APA events data was excluded if: (i) the rate of NA values is >95% and (ii) the standard deviation between samples of a given gene is <0.05. The significance was set as $P < 1 \times {{\mathrm{e}}}^{ - 5}$. The age and sex of patients were set as covariates. Only the overlap results of two methods will be retained in the database. Details of the methods were summarized in the Supplementary Table S3.

### Identification of genetic variations that affect APA events

The canonical PAS sequence (AAUAAA) or its variant sequences (i.e. AUAAAG, AAAAAA, AUAAAA and AAAAUAA) are defined as critical upstream signals of APA (2). Single nucleotide polymorphisms (SNPs) can regulate APA events through PAS and affect the risk of multiple diseases (22). In the present study, we extracted SNPs located within 40-nt upstream of the PAS using BCFtools (v1.10.2) (22). We then used BEDtools (v2.30.0) to extract sequence information 5-nt upstream and 5-nt downstream of SNPs (23). We annotated these SNPs into three categories according to the effects on PAS, including creation of a novel PAS (shown as ‘create’ in the database), interruption of a PAS (shown as ‘interrupt’ in the database) and change from one PAS to another (shown as ‘change’ in the database). Then, we performed ANOVA analysis to compare the PDUI between different SNP genotypes. The threshold was set as *P* < 0.05.

Additionally, extensive evidence has shown that RBPs play critical roles in APA regulation (24). Thus, we also identified the SNPs in RBP genes and their effect on APA events of RBP targets. Then, we then performed ANOVA analysis on the PDUI of RBP targets between different SNP genotypes. The threshold was set as *p*<0.05.

### Quantitative trait methylation analysis of APA (apaQTM)

A previous study has reported that DNA methylation is a potential regulator of APA. For example, in the absence of DNA methylation, CCCTC-binding factor (CTCF), can bind to regions downstream of proximal poly(A) sites, which further promoting proximal poly(A) isoform expression in colon cancer cells (25). Therefore, in this study, we identified the associations between DNA methylation level and APA events by using methylation quantitative trait loci (also called quantitative trait methylation, QTM). Firstly, the Illumina Human Methylation 450 data of samples across 32 cancer types were downloaded from TCGA using firebrowse (http://firebrowse.org/). We first performed quality control for the methylation data. The methylation data was excluded if: (i) the rate of NA values is >95% and (ii) the standard deviation between samples of a given methylation data is smaller than 0.05. Total 3 760 315 methylation events were excluded during the quality control step. Then, same as the QTL analysis, we identified the effect of DNA methylation on APA events using additive linear model in MatrixEQTL package. The age and sex of patients were set as covariates. The pairs with p-value less than $1 \times {{\mathrm{e}}}^{ - 5}$ were identified as methylation–APA pairs.

### Drug information on APA genes

To identify the related drug information for APA genes, we obtained information of drug-target gene interactions from DrugBank database (v5.1.9), including gene symbol, Uniprot ID, DrugBank ID, Drug name, Drug type and Drug status (26). Then, we searched each APA gene in drug-target interaction profile to identify potential drugs for APA genes. If the searching result shows duplicated, only one record will be left in our database. Drugs provided in the database were grouped using Anatomical Therapeutic Chemical (ATC) classification system codes. Detailed information of data analysis steps was summarized in the Supplementary Table S3.

### Manual curation of PubMed articles

We searched publications in PubMed for 10 363 APA genes. Taking CTNNB1 as an example, the keywords used for searching were as follows: ‘CTNNB1[Title/Abstract] AND alternative [Title/Abstract] AND polyadenylation [Title/Abstract]’. After a manual review of the paper, we selected 135 literatures related to the function of APA events in corresponding cancers.

### Database construction

CAFuncAPA is freely available at https://relab.xidian.edu.cn/CAFuncAPA/. The front-end of CAFuncAPA website was developed using Vue 3.0 and Element Plus (https://element-plus.org/). The back-end of the website was developed using node.js and express (https://expressjs.com/). Data storage and management were performed using MySQL v5.7 (https://www.mysql.com/). All upstream and downstream analyses were performed using R 4.0.3 based on the Linux system. All data in CAFuncAPA can be download on the website. CAFuncAPA website supports popular web browsers, such as Google Chrome, Firefox, Safari, and Microsoft Edge.

## DATABASE CONTENT AND FEATURES

### Overview of CAFuncAPA

**Figure 1.**
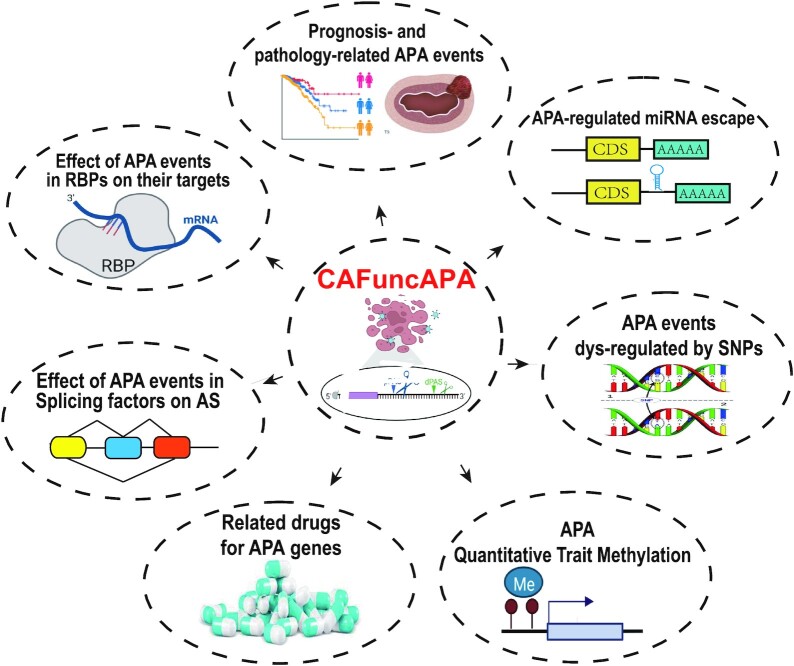
Content of CAFuncAPA. CAFuncAPA contains five APA downstream effects and two up-regulator analyses. The downstream effects included analyses of APA effects on genes/isoforms expression, miRNA binding regulation, RNA binding protein (RBP) regulation and alternative splicing regulation. The up-regulator analyses included the effect of the genetic variants on APA events (genetic variants on PAS and RBP genes), and the effect of methylation phenotypes on APA events.

CAFuncAPA aims to provide a resource for intensive APA functional annotation in human pan-cancer. Figure 1 shows the content and construction of CAFuncAPA. CAFuncAPA provides multiple unique functions for APA regulation in pan-cancers. Based on APA events across 32 cancer types collected from TC3A database, we provide several functional annotations that didn’t covered by previous databases. For example, we annotated the potential down-stream effects of APA into four parts, including APA effects on genes/isoforms expression, miRNA binding regulation, RBP regulation and alternative splicing regulation. Moreover, we investigated the effects of potential APA up-regulators, including the effect of genetic variants on PAS, the effect of genetic variants in RBP genes on APA events, and the effect of DNA methylation phenotypes on APA events (Supplementary Table S1). These novel functions provide by CAFuncAPA fill the gap of the potential regulation of APA in pan-cancer.

To construct the database, firstly, >15 000 APA events across 32 cancer types were collected from the TC3A database. Based on the survival analysis and ANOVA analysis, we identified 2549 survival-associated and 2946 stage-associated APA events. Correlation analysis between tumor-associated APA events and genes/isoforms expression identified 1785 APA events that potentially regulate the genes/isoforms expression. Identification of APA-regulated miRNA loss found 3328 APA-mediated miRNA losses, leading to the altered expression of 9801 target genes. Interaction analysis of APA events on RBP genes and RBP targets identified 85 APA-regulated RBP genes, which potentially affect 17 543 tumor-specific target genes and 85 common target genes in more than five cancers. Interaction analysis of APA events on splicing factors and AS identified 44 APA-regulated splicing factors, which potentially affect 8983 tumor-specific AS events and 424 common AS events in more than five cancers. Annotation of functional SNPs on PAS identified 13 PAS directly affected by SNPs leading to significant APA alteration. Interaction analysis of SNPs on RBP genes and APA on RBP targets identified 7618 APA events that are potentially affected by SNPs. apaQTM analysis identified 63086 APA events that are potentially affected by DNA methylation. Supplementary Table S4 shows the number of annotated APA events in each functional analysis category.

For example, one significant APA biomarker on Catenin Beta 1 (CTNNB1) in kidney renal clear cell carcinoma (KIRC) was found to upregulate CTNNB1 gene expression (Figure S1A–C), which was consistent with a prior study (27). This may be caused by its interference with the inhibition of several miRNAs, such as miR-330–3p, according to the miRNA results (Figure S1D). Due to the preference of this gene to use the proximal site, and the higher CTNNB1 expression associated with poorer survival, this APA event may be a prognostic marker in KIRC patients (Figure S1A and C). In contrast, escaped miRNAs may negatively regulate the expression of tumor suppressor genes, such as RBX1, to further promote cancer progression (Figure S1D) (28). The present results suggested CTNNB1-miR-330-3p-RBX1 as an APA-induced mechanism for KIRC progression. Moreover, drug analysis identified CTNNB1 as a druggable target that showing interaction with Urea (Figure S1E). As shown in here, CAFuncAPA can be used to identify the potential functions of APA events and the underlying mechanism of APA regulation in human cancer. All related files in CAFuncAPA are freely available and can be downloaded on the ‘Download’ page.

### Annotations and utility of CAFuncAPA

#### The gene structure browser provides information on tumor-associated APA events

**Figure 2.**
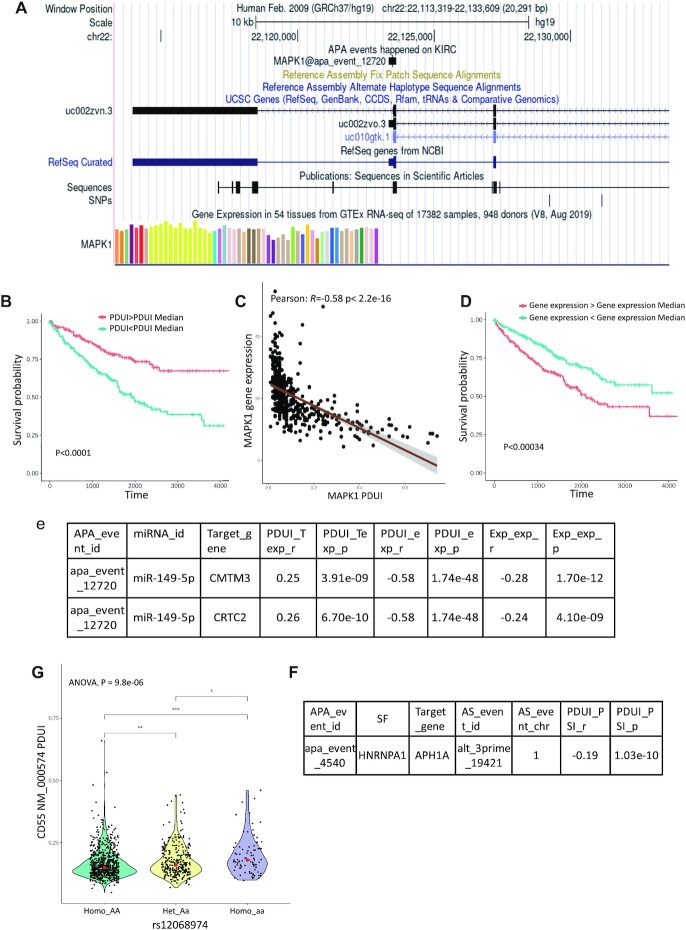
Example of functional analyses of CAFuncAPA. (**A**) Genomic structure of APA events of TCGA. Genome browsers also provide transcript isoform and other structural information such as SNPs. (**B**) K–M survival analysis showed that APA event in MAPK1 was associated with KIRC survival. Patients with higher PDUI had better survival than patients with lower PDUI. (**C**) Correlation analysis between APA events and the expression of MAPK1. A negative correlation showed that a lower PDUI (shorter 3’UTR) was significantly correlated with higher gene expression. (**D**) The expression of MAPK1 showed a significant association with KIRC survival. (**E**) Escaped miR-149-5p may potentially inhibit CMTM3 and CRTC2, resulting in an oncogene-miRNA-suppressor axis in cancer. (**F**) APA event on HNRNPA1 in breast cancer may potentially affect the AS event on APH1A. (**G**) Different genotypes of HNRNPU showed an association with APA events on CD55 in breast cancer.

To understand the landscape of APA events in cancers, the co-localization of APA with reference gene structure information is necessary. Here, we projected each APA event on the gene structure track provided by the UCSC genome browser (29). Taking mitogen-activated protein kinase 1 (MAPK1) as an example, MAPK1 has two cancer-specific APA events in kidney renal clear cell carcinoma (KIRC), including ‘apa_event_4877’ and ‘apa_event_12720’. As shown in Figure 2A, the predicted proximal site of apa_event_12720 located at 22123458 on chr22, which may be an APA event related to a transcript isoform of MAPK1 (isoform ID: uc002zvo, location: 22123319–22123609). apa_event_4877 was located on another transcript isoform (isoform ID: uc002zvn, location: 22113947–22118529). For each gene, users can obtain a detailed annotation of APA event information, APA-related transcript isoforms, and other structural information such as SNPs, and motif region by ChIP-seq extracted from the ENCODE database (Figure 2A) (30). As shown here, the APA event gene structure browser will help users identify cancer-specific APA events and obtain basic information on these APA events.

#### APA-regulated genes/isoforms expression may affect prognosis and pathology stages

Previous studies have demonstrated that APA-regulated gene expression alterations have a strong prognostic power for cancer patients (31). To identify APA events affecting prognosis and pathology stages through gene/isoform expression, we performed correlation analysis between prognosis- and stage-associated APA events and gene/isoform expression profiles. We identified 1785 APA events that potentially regulate gene/isoform expression. We found that the APA event (apa_event_12720) of MAPK1 in KIRC was significantly associated with patient survival (Figure 2B). The group with a higher PDUI (longer 3’UTR) had a significantly better prognosis than the group with a lower PDUI (shorter 3’UTR) (*P* < 0.0001). Moreover, the APA event of MAPK1 was strongly correlated with MAPK1 gene expression (*P* < 2.2e–16 and *r* = –0.58), which indicated APA-medicated 3’UTR shortening may potentially result in MAPK1 overexpression (Figure 2C). Additionally, the expression of MAPK1 is also a prognosis-related biomarker. As shown in Figure 2D, higher MAPK1 expression was also associated with poor prognosis in KIRC patients. Our results were consistent with those of previous studies (32,33). MAPK1 has been identified as a critical oncogene that contributes to tumorigenesis, metastasis, and angiogenesis through MAPK signaling pathways (32).

#### APA-mediated miRNA escape may lead to oncogene-miRNA-suppressor axes in cancer

APA-mediated 3’UTR changes can cause the inclusion or deletion of miRNA binding sites, which has a wide range of effects on mRNA expression. During the tumorigenesis process, proto-oncogenes start to use proximal poly(A) sites and generate shortened 3’UTR, which promotes mRNA escape from miRNA inhibition. Here, we identified 3519 APA-mediated miRNA losses using TargetScan. Moreover, escaped miRNAs may bind to other target genes and lead to decreased expression of target genes. A previous study has suggested that the oncogene-miRNA-suppressor axes may be common participants in tumorigenesis (34). For example, we found that three miRNAs may be lost due to the APA event on MAPK1, including miR-149-5p, miR-224-5p and miR-3064-5p (Supplementary Table S5). According to correlation analysis between APA genes and miRNA target genes, we found that 79, 32 and 94 target genes may potentially regulate by miR-149–5p, miR-224-5p and miR-3064-5p, respectively. Among them, we found the expression of several tumor suppressor genes that showed a negative correlation with MAPK1 expression, such as CMTM3 and CRTC2 (35,36) (Figure 2E). These results suggested that APA-mediated miRNA loss may be the underlying mechanism that results in the activation of oncogenes and inhibition of tumor suppressor genes in cancer.

#### APA events on RBP genes may have a potential effect on RBP target genes

RBPs are critical regulators of APA decisions (3). Thus, expression changes in these RBPs can cause differential cleavage and polyadenylation outcomes. Interestingly, previous study have found that RBP genes themselves also undergo APA (24). Changes in RBP gene transcripts may result in alterations in target gene expression and stability. In this work, we identified the effect of APA events on RBP genes on RBP targets using both correlation analysis and QTL analysis. We identified APA event on Serine and Arginine Rich Splicing Factor 7 (SRSF7) in breast cancer (apa_event_918) (2). SRSF7 is also identified as a splicing factor, which constitute part of the spliceosome (37). We found 3150 targets to be regulated by SRSF7 (Supplementary Table S6). The APA event on SRSF7 may potentially regulate the expression of ABAT according to our results. Previous studies have implicated that the expression of ABAT is a prognostic marker in breast cancer (38). Thus, these findings indicate how this analysis will help users identify APA events on RBPs and their potential effects on RBP target genes.

#### APA events on splicing factors may have a potential effect on alternative splicing events

A previous study has suggested that APA-dependent function of SFs play important roles in cellular senescence and cancer (39). Moreover, differential PAS selection on SFs indicated that APA may be involved in the regulation of alternative splicing (21). To identify APA-regulated SFs and AS, we performed correlation analysis between APA events on SFs and AS events on target genes. In total, 44 APA-regulated SFs were identified to have a potential effect on 8983 tumor-specific AS events. For example, we found an APA event on HNRNPA1 in breast cancer (apa_event_4540), and it may potentially affect the AS event on APH1A (Figure 2F). An AS event on APH1A has been identified a predictor in breast cancer (40). Therefore, this analysis provides APA-AS interactions across cancers, which will help users explore the APA regulation mechanisms on AS events through splicing factors.

#### APA events may potentially be affected by single nucleotide polymorphisms (SNPs)

Previous studies have demonstrated that some single nucleotide polymorphisms (SNPs) dysregulate PAS to contribute to tumorigenesis (22). Here, we systematically identified SNPs that regulate PASs and affect APA events across cancers. We identified nine SNPs that interrupted the PAS, and four SNPs created a novel PAS (Supplementary Table S7). Moreover, to better understand the indirect effect of SNPs on APA events, we identified SNPs on RBP genes that may potentially affect APA events on RBP targets. Here, we identified 1989 SNPs on RBPs that may potentially affect 7618 APA events. For example, as shown in Figure 2G, we found that rs12068974 was located on HNRNPU, and may potentially affect APA events on CD55 in breast cancer. HNRNPU is a critical regulator of APA decisions (41). The alteration of HNRNPU may lead to distinct APA events on target genes. A previous study has demonstrated that HNRNPU may bind to CD55 mRNA (42). Altered CD55 expression has been identified as a significant biomarker in breast cancer (43).

#### APA-associated apaQTM pairs

DNA methylation and APA dysregulation are both highly relevant to cancer. A prior study has identified a methylation-regulated APA mechanism, demonstrating that DNA methylation regulates APA events (25). Therefore, we performed MatrixEQTL to identify methylation-APA pairs across cancers. Using this approach, we identified 38 188 apaQTM significant pairs with a significance level $P < 1 \times {{\mathrm{e}}}^{ - 5}$. With CAFuncAPA, users can obtain information on methylation such as composite and chromosome locations, and the statistics of apaQTM pairs, such as *P*-values and beta values.

#### APA gene-related FDA approved-drugs

CAFuncAPA also provides pharmacological information extracted from DrugBank for genes with APA events. We identified 4015 drugs for APA genes. For a given gene, related drugs, drug activity, drug type and drug status are provided. For example, MAPK1 is a druggable gene that can interact with 17 small molecule drugs, such as hypothemycin and perifosine (Supplementary Table S8). As shown here, this module will provide potentially druggable targets for anticancer studies.

## DISCUSSION

**Figure 3.**
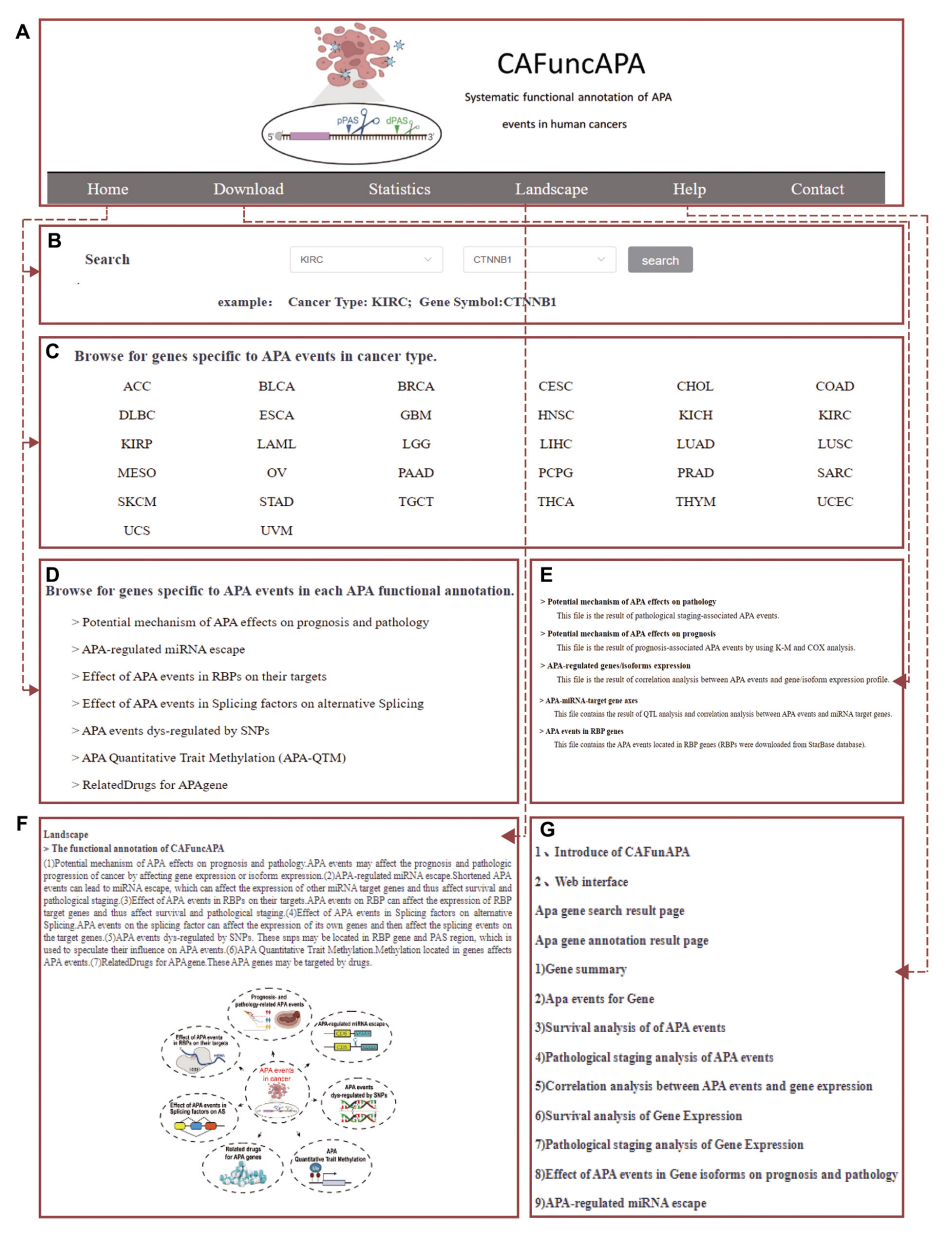
The main functions of CAFuncAPA. (**A**) The logo and the top navigation bar of the main functions in CAFuncAPA. (**B**) ‘Quick search’ function of CAFuncAPA. Users can choose the cancer type and the gene symbol of interest. (**C**) Cancer type included in CAFunAPA. Users can obtain cancer-specific APA genes through this function. (**D**) Functional analyses included in CAFuncAPA. Users can obtain APA genes related to each functional annotation. (**E**) The Download function in CAFuncAPA. (**F**) The Landscape of functional annotation analyses in CAFuncAPA. (**G**) The Help function contains a brief introduction of CAFuncAPA and its functions.

CAFuncAPA is the first database that systematically annotates diverse APA functions in human cancers from diverse aspects (Supplementary Table S1). The current version of CAFuncAPA contains seven functional annotations for 15 478 APA events across 32 cancer types. Moreover, CAFuncAPA provides a user-friendly searching and browsing interface. As shown in Figure 3A and B, users can conduct quick data queries on the ‘Home’ page by selecting the cancer type and gene symbol of interest. Users can also browse all cancer-specific APA genes by clicking the cancer type (Figure 3C). Additionally, all functional analyses are also available separately on the home page (Figure 3D). CAFuncAPA also provides downloadable results, statistics and help functions (Figure 3E–G).

To make better use of CAFuncAPA, we plan to add functional analysis results for APA events from cell line data and single cell sequencing data in the future. Moreover, we will continue to expand our analysis and continue searching for experimentally validated APA events and their functions in cancers. We believe that CAFuncAPA can be routinely used in cancer studies for a better understanding of APA regulation and functions of oncogenes in cancer biology.

## DATA AVAILABILITY

All data in CAFuncAPA can be download on the website (https://relab.xidian.edu.cn/CAFuncAPA/).

## Supplementary Material

zcad004_Supplemental_Files
