# Supplementary material for: CAFuncAPA: a knowledgebase for systematic functional annotations of APA events in human cancers
Source: NAR Cancer. 2023 Jan 23;5(1):zcad004. doi: 10.1093/narcan/zcad004 (PMC9869079; doi:10.1093/narcan/zcad004)
Supplement: zcad004_Supplemental_Files [file zcad004_supplemental_files.zip › Supplementary Figure.pdf]

Supplementary Figure

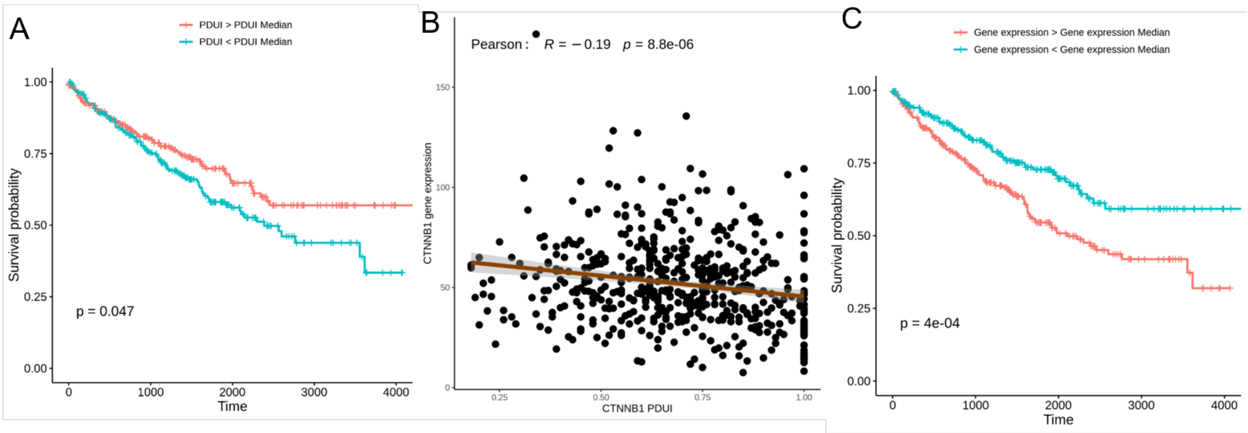

D

APA_event_id	miRNA_id	Target_gene	PDUI_Texp_r	PDUI_Texp_p	PDUI_exp_r	PDUI_exp_p	Exp_exp_r	Exp_exp_p
apa_event_1479	miR-330-3p	RBX1	0.42	1.94e-24	-0.21	1.64e-06	-0.42	4.56e-28

E

Gene	UniProtAcc	DrugBank ID	Drug name	Drug activity	Drug type	Drug status
CTNNB1	P35222	DB03904	Urea	Catenin beta-1	small molecule	Approved | Investigational

Figure S1. An example of APA biomarker on Catenin Beta 1 (CTNNB1) found in CAFuncAPA. (A) The APA event on CTNNB1 showed prognostic value in KIRC patients. (B) The correlation between the APA event on CTNNB1. (C) The expression level of CTNNB1 showed prognostic value in KIRC patients. (D) APA on CTNNB1 may lead to loss of miR-330-3p binding sites. Escaped miR-330-3p may potentially bind on tumor suppressor gene RBX1. (E) drug analysis identified CTNNB1 is a druggable target that showed interaction with Urea
